# Sources of information used by elite distance running coaches for selection decisions

**DOI:** 10.1371/journal.pone.0268554

**Published:** 2022-08-08

**Authors:** Kathryn Johnston, Joseph Baker

**Affiliations:** Kinesiology and Health Science, York University, Toronto, Ontario, Canada; University of Calgary, CANADA

## Abstract

Talent identification and selection are critical components of competitive sport success. Despite the time, effort, and resources invested, the accuracy of selection decisions remains generally poor. While much of the scholarship in this area has focused on the factors discriminating skilled and less-skilled individuals, limited research exists on *what* information is used in the decision-making process for athlete selection. The current study seeks to gain a better understanding of the information used by elite distance running coaches when forming judgements for athlete selection. Ten semi-structured interviews with elite distance running coaches from across Canada were transcribed and analyzed using inductive thematic analysis. It was interpreted that coaches mainly gather information using their coach’s eye to determine an athlete’s ‘fit’ to the team. Coaches also use more objective information such as race times and movement analyses to assess performance and judge future ‘potential’. As well, the decisions were believed to be influenced by situational considerations at the time of the selection procedure. Specifically, these considerations affecting a coach’s selection included length of time to make a decision, personal limitations in decision-making abilities, and team circumstances. Interestingly, coaches recognized limitations in their selection practices and procedures and discussed some of their personal and system-level biases, highlighting their awareness of potential selection inefficiencies/inaccuracies. Overall, distance running coaches used a variety of techniques to gather information before a selection was made, relying on both subjective and objective information for crafting judgments. Findings are discussed in relation to implications for coaches, sport organizations, and talent identification and selection programs.

## Sources of information used by elite distance running coaches for selection decisions

Evidence-based approaches to talent identification and selection have become critical components of sport systems around the world, reflected in the integration of sport science experts (analysts, medical teams, and researchers) and an increased use of technology [1,2]. There has recently been a large upswing in the number of scientific articles on talent identification [3], especially on the assessment of the efficacy of selections between skilled and less-skilled athletes [4–6]. A strong research bias has been observed towards physical testing, speaking to a limited understanding of the ‘less-tangible’ characteristics of elite athlete performance [3,7]. Despite the common use of physical measures, it is important to acknowledge that it is unlikely coaches rely on such a narrow set of attributes for talent selection. Given the multidimensionality of sport (i.e., requiring tactical, inter-/intra-personal, perceptual-cognitive skills etc.), it is more likely coaches use (either consciously or subconsciously) a more complex approach to athlete selection than has been studied in prior research. In reality, most coaches and organizations appear to use a variety of information sources for identifying skilled performers (often referred to as ‘talent’) [8–10]. However, little is known about what that information is and how that information is used in the decision-making process for athlete selection.

To investigate this, Roberts and colleagues [11] recently conducted a systematic review on the types of information coaches use for ‘talent’ selection decisions. Their review highlighted 14 studies revealing four main types of information. Specifically, when coaches were asked how they identify ‘talented’ athletes, ‘gut feeling’ or ‘instinct’ (also known as the coach’s eye), was the preferred method of identification for many (see Christensen, [12]; Holt & Dunn, [13]; Johansson & Fahlén, [14] for specific articles). Other themes included ‘drive and ambition’, ‘game intelligence’, and ‘physical and technical skills’.

In addition to the work by Roberts and colleagues on the types of information used by coaches for ‘talent’ selection, MacMahon and colleagues [15] explored the factors affecting athlete selection (which they termed ‘recruitment’). Using semi-structured interviews, the researchers identified factors influencing a group of recruiters’ (who were responsible for scouting prospective players) decisions for athlete selection within the Australian Football League’s (AFL). These factors included a) recruiter background, b) recruiter attributes, c) recruiter understanding of team needs, and d) recruiter-coach relationship. Their work revealed both intuition and deliberation are used by AFL recruiters, and the extent to which they are used appears to be strongly influenced by the recruiter-coach relationship. This finding speaks to the complex (many inter- and intra-personal factors at play), dynamic (in a state of change/flux), and interconnected (considers the relationship between the selector and athlete) nature of athlete identification and selection.

This complexity is represented in the work done by Christensen [12], (and further supported empirically by Roberts et al., [16]), who hypothesised two different coaches will identify ‘talent’ differently. Recently, Jones and colleagues [17] also noted this finding, and concluded coaches conceptualize sporting ‘talent’ in different ways within different contexts. With this in mind, *who* is performing the identification may be just as important as *what* is being identified [18]. It is important to acknowledge, however, coaches do not only try to select the ‘best’ or most ‘talented’ athletes, they also make decisions based on their circumstances (i.e., the amount of information accessible and the context it is situated within) [17]. However, this type of complexity expressed by coaches during the decision-making process is rarely acknowledged or considered in the research. Often, this leads to a reductionist perspective of what athlete selection looks like (i.e., heavily dominated by physical assessment), which exposes the difficulty in determining how and why coaches craft judgements and make selection decisions the way they do. In their work, Cushion [19] and Mills and colleagues [20] acknowledged this ‘bioscientifically-dominated’ perspective and encouraged researchers to adopt a multidimensional lens that considers the athlete beyond his/her/their physicality and to explore the broader social influences at play. If research endeavours are able to gain a better understanding of coaches’ and athletes’ unique, experiential knowledge, it is plausible this information could enhance coach education tools and thus, enhance coach-athlete interactions [21,22]. To this end, the objective of the present study was to better understand coaches’ experiences with athlete selection practices and to illuminate the sources of information coaches use when forming judgments and making selection decisions.

Specifically, this work seeks to better understand selection practices in the unique domain of distance running in Canada. There are multiple pathways an athlete can take to reach the highest levels of competitive distance running in Canada; however, the primary route would include private club-based participation and school-based (i.e., college or university) participation. Both of these avenues funnel into Canada’s provincial and national sport organizations, where resources are streamlined to support an athlete’s journey to international competitions. Canada has a relatively long history with track and field and athletics, as the national governing body (Athletics Canada) is one of Canada’s oldest National Sport Organizations (established in 1884) [23]. With training centres spanning coast to coast, Athletics Canada has a purpose “to support high performance athletics excellence at the world level, and to provide leadership in developmental athletics” [23]. Coupled with an increase in funding and scientific attention, (e.g., government funding has more than tripled in the past 15 years [24]), Athletics Canada has seen improvements in podium finishes at major events like the Olympics and Commonwealth Games over the past two decades [25]. Although Canada is not necessarily known for developing world-class long-distance runners (compared to nations like Kenya and Ethiopia), multiple Canadians (males and females) have rankings in the top 100 athletes in the world for races such as the 5000 meter, 10,000 meter, half marathon, and marathon (as of Jan 4 2022 [26]).

Perhaps in response to the international success and accolades, Canadian scientists and writers (for examples see [27–31]) are exploring this ‘subculture’ of distance running within athletics, as it has been recognized as a ‘uniquely social world’ p. 387 [32]). Among other things, this subculture is renowned for placing value on certain traits such as the ability to tolerate and even embrace the routineness of pain, combined with extensive and exhaustive training [27,32–34]. These values present an interesting area for exploration from both an athlete level (e.g., examining personal identity [32,35,36]) and from the perspective of coaches to illuminate what qualities, characteristics, and traits are valued and selected for within the Canadian sport landscape. To the best of the authors’ knowledge, this is the first study of its kind to unpack the selection decisions from the perspective of elite sport coaches within the Canadian context. While work by other Canadian researchers [28,37] have sought to explore the ways in which coaching practices have been shaped through social, historical, and cultural contexts, the present study seeks to explore the experiences with selection practices within this subculture of coaches.

## Methodology

The present investigation was part of a larger research initiative examining talent and athlete selection in elite distance running and uses the same data corpus. The first study [38] examined coaches’ subjective beliefs of the term ‘talent’ in the context of distance running. While the first study examined theoretical constructs and definitions, the current investigation focuses on the approaches, beliefs, and practices regarding athlete selection. We recognize this is not an ideal approach and raises concerns about ‘data slicing’, however we believe a) the interviews provided such rich conversation that condensing both research questions into one manuscript may lead to an overly generalized view of the data, b) there was minimal overlap between the material presented in Study 1 and the current study, c) the authors designed the study with the intention to gather two sets of different data using two separate research questions with this very hard to access sample, and thus, believe the dataset is worthy of supporting multiple, distinct papers [39], and d) there is precedent for this type of approach (see [40–42]).

### Theoretical perspective

To guide our study, we draw upon the ontological and epistemological principles and philosophies relating to pragmatism. Pragmatism is a suitable paradigm for the present research given the debate about conceptualizations and interpretations of reality and that notions of reality should be examined using various research tools that help to answer a question and/or solve a problem. In this sense, the authors subscribe to the notion that multiple research approaches can and should be used to help answer the question ‘how do coaches select athletes?’. Specifically, this research seeks to understand how coaches select athletes using one specific analytical approach (qualitative description), but this area would benefit from other approaches (both qualitative and quantitative) to gain a more thorough understanding.

While different versions of pragmatism exist (see [43] for an example) the present study draws on more traditional and classical versions of pragmatism, which accept people’s ideas and beliefs as tools for problem solving [44]. From this perspective, examining the experiences shared by elite coaches when selecting athletes provides an indication of what has ‘worked’ in the past. While this ‘what works’ perspective is criticized by some theorists (see [45]), others argue this perspective has the potential to help researchers map out various processes and procedures to guide social action. This classical view also respects what has ‘worked’ in the past does not assure what will ‘work’ in the future [46]. Therefore, the present investigations may hold value for coaches in particular contexts and may be of value for the creation of knowledge tools (such as coach education content); however, findings should not necessarily be interpreted with a prescriptive lens.

This practical, and ecological view of knowledge is a keystone feature of pragmatism [44], and one that is particularly valuable in this context. For example, when writing the analysis and discussion, pragmatism influenced the type of approach and techniques used to assess, analyze, and interpret the data. Similarly, through this pragmatistic lens, the authors focused the examination of the data on actionable knowledge (in a similar sense to the work of Kelly and Cordeiro [44]), which further helped to distill key takeaways for what has ‘worked’ for coaches in the past in relation to athlete selection.

### Analytical foundation

A prominent theme in the literature on pragmatism is that it provides a framework to help researchers choose which methods will be most appropriate rather than dictating a certain methodological approach [47–49]. The analytical approach chosen for the present study is qualitative description (QD). This approach is appropriate for both the philosophical position and the research question under investigation because the overarching goal of QD is to describe an individual’s experiences (on a surface level) with the hope of transforming participants’ ideas, themes, or concepts and developing them into educational or behavioural intervention strategies [50–54]. This approach allows researchers to stay ‘close’ to the data (using low inference) and seeks to report on the participant’s experiences, without trying to discover the essence and meaning of those lived experiences (as in approaches such as descriptive phenomenology [50]). QD is a practical choice for studies that seek to investigate a description of the phenomena, and while much of the QD work is applied to health care research [52,55], the approach has been promoted by researchers to expand to other domains [53]. QD is particularly relevant for exploring the present research question as the literature on ‘talent’ and ‘talent selection’ practices is quite sparse, and the scholarship could benefit from both surface level reporting (using QD) and thick description (as found in other qualitative approaches like ethnography) to broaden the scope of the research.

### Participants

After obtaining university research ethics board approval (University Research Ethics Board certificate number for approval: STU 2019–067), participants were contacted via email to gauge interest in the project. Initial participants were contacted using the authors’ personal contacts, and thereafter, snowball sampling and peer recommendation approaches were employed. Once written consent was obtained, an interview was arranged (either in person, over Skype, or over the phone). Interviews were conducted with 10, male coaches from across Canada who were either presently or previously working with distance running athletes (racing distances between 5km and 42km). The coaches worked with athletes at various competitive levels; regional (n = 1), national (n = 5), or international/Olympics (n = 4) and had various years of experience competing themselves, ranging from regional- to Olympic-level competition. Many coaches held multiple coaching roles or coached athletes at different competitive levels at the same time (e.g., university-level, provincial- and/or national-level athletes). All but one of the coaches were actively coaching/working with athletes at the time of the investigation. To protect the coaches’ anonymity, numerical codes have been used (Coaches 1–10) along with pseudonyms for any location and/or athlete the coach may have named during the interview.

### Interview guide

A key tenet of QD research is using expert knowledge of key informants to focus on an area that is under explored [50,51]. Knowing this, the authors met with two leading researchers in the field of talent in sport and two practitioners in distance running and endurance sports to discuss the nature of the questions being asked and the way the questions were framed in the context of the broader research goals. Additionally, the interview guide was piloted with a group of nine collegiate level coaches and questions were modified to enhance the fluidity, interview length, and foci of the questions. The length of the interviews ranged from 24 minutes to 2 hours and 36 minutes with an average time of 36 minutes. The primary questions guiding the discussion along with a sample of probing questions to gain a deeper understanding about the coaches’ selection processes and decision-making strategies has been added in Table 1.

**Table 1:** Interview guide.

Main question	Potential probing question(s)
Can you tell me a little bit about how you arrived in this coaching role?	How long have you been coaching? What level of competition do you coach right now? What is your current role working with athletes? Have you held other roles while working with athletes in the past?
In your role(s), can you tell me your relationship with assessing athletes’ abilities and making selection decisions?	Are you directly responsible for making selection decisions? How often would decisions be made? Is anyone else making this decision with you? Please explain who and how they influence the decision process
Can you walk me through your assessment and selection processes and procedures?	When does the assessment procedure start? What types of assessments do you use? Physical assessment? Psychological assessments etc.? Has this approach changed over the years? If so, how, if not, why do you think that is?
What are the specific aspects you are looking for during assessments?	Are there certain physical, social, etc. components?
How long does it take to make a selection decision?	Is this something that is obvious or more difficult to tell?
Is there anything else you would like to share about your experiences when assessing athletes and making selection decisions?	

### Positioning the authors in the research

The authors have been involved with athlete selection discussions with practitioners in the field and tried to position themselves within the data collection approaches in a way that reflects and embraces those experiences. Specifically, both authors have held consulting positions with a variety of high-performance sport organizations involved in improving identification practices and minimizing ‘talent wastage’ within selection systems. For this reason, the authors’ experiences in athlete selection practices likely influenced the way the research questions, study design, analysis, and reporting were conceived and executed. Fundamentally, the authors’ choice of pragmatism as the overarching philosophical orientation was influenced by our desire to understand selection practices from the experiences of coaches with the hopes of building the foundational knowledge for coach education. This has likely come from a recognition that coaches’ voices regarding their experiences in athlete selection processes, combined with research and education on this topic, were lacking from a research perspective.

### Data analysis

The method used for data analysis was Inductive Thematic Analysis (ITA). ITA was particularly useful for this study because the literature to date indicates a variety of styles, practices, and approaches are used in selection, and thus, agreed upon, pre-determined themes do not exist in the literature to the best of the authors’ knowledge. The analysis process was shaped by the suggestions of Braun and Clarke’s [56,57] work, with the initial step of data familiarization (where the researchers listened and re-listened to the interviews and read and re-read the transcriptions). Following this step, the researchers looked for commonalities and differences in the coaches’ experiences and notes were made to capture general ideas. Thereafter, an initial set of codes was created and assigned to meaningful statements identified by the researchers. These codes were used to generate an initial set of themes from the data, and the researchers then embarked on a process of checking, re-checking, naming and re-naming the themes (in this sense, a ‘theme’ is used to capture “patterns of shared meaning, united by a central concept or idea” p. 342 [58]), This ‘open coding’ process did not utilize a coding framework, rather an interpretive reflexive process (i.e., *reflexive TA*), which allowed the themes to become the ‘outcome’ and thus the foundation for the discussion [58]. This method allows the data to evolve instead of forcing it into neat, pre-determined themes, which is well-aligned with the principles of QD [59]. To help demonstrate the coding and theming process, see Table 2 which shows specific codes and themes and the role they played in the analysis.

**Table 2:** Example of interview coding.

Example meaning unit	Example code	Example category	Theme
We’ve had athletes, who you know say “[Coach 10], I’m gonna go Uber to Walmart and get some ponchos for the girls” or something. You know?…That’s great. Or bringing snacks for everybody or, I mean, those are all little things but you know, if there’s a bunch of things like that, that’s really helpful to the team dynamic right or you know, I don’t know the girl that braids all the other girls’ hair right or always brings the ribbons or you know, brings the face tattoos or whatever.	Recognition of ‘softer’ skills	Characteristics for selection	Subjective criteria and the coaches’ eye
Thankfully haven’t had to make too many really tough ones [selection decisions] usually is pretty clear cut with head to head racing and you know within the season, umm it’s, it’s a previous performance we’re talking about from an upper year student and how they performed in the championships	Displays of performance	Selection decision-making	Objective information
If there’s eight runners who you know can stay together on a workout or run and can work together, but that ninth person is just significantly off the pace and can’t can’t hang together just for logistics reasons, I often wont invite them out. You’re managing people out on roads and through the city and in the trails and you can’t have people getting left behind or dropped.	Consideration for logistics	Selection decision-making	Team circumstance

### Trustworthiness of the data

Specifically, the authors sought to increase the credibility of the study by utilizing appropriate and rigorous data collection, data management, and data analysis procedures outlined by leading researchers in the field [50,56]. In line with reflexive TA principles, author notes were created throughout the data analysis process. These notes were not only shared with the second author, but also with other researchers and practitioners in the field (those who were invited to contribute their perspective on the interview guide). The use of these notes and note-sharing practices helped minimize the influence of the lead author’s personal experiences when interpretating the coaches’ comments [60].

## Results

Broadly speaking, the coaches in this sample employed a multidimensional approach when gathering information for selection decisions. For example, when asked what information he uses to select athletes, Coach 1 stated:

We have multiple processes; we have a rank list, or a list we would love to talk to and love to recruit that we’ve identified based on their times, national team appointments, connections with coaches, etc. That would be part one; within that, were looking at training age, training history, chronic training load, multi-sport—like when did they end multisport, how many years have they been specialized, what other sports were they doing, is there talent transfer that comes over to that, personality, culture fit, things like that.

**Fig 1:**
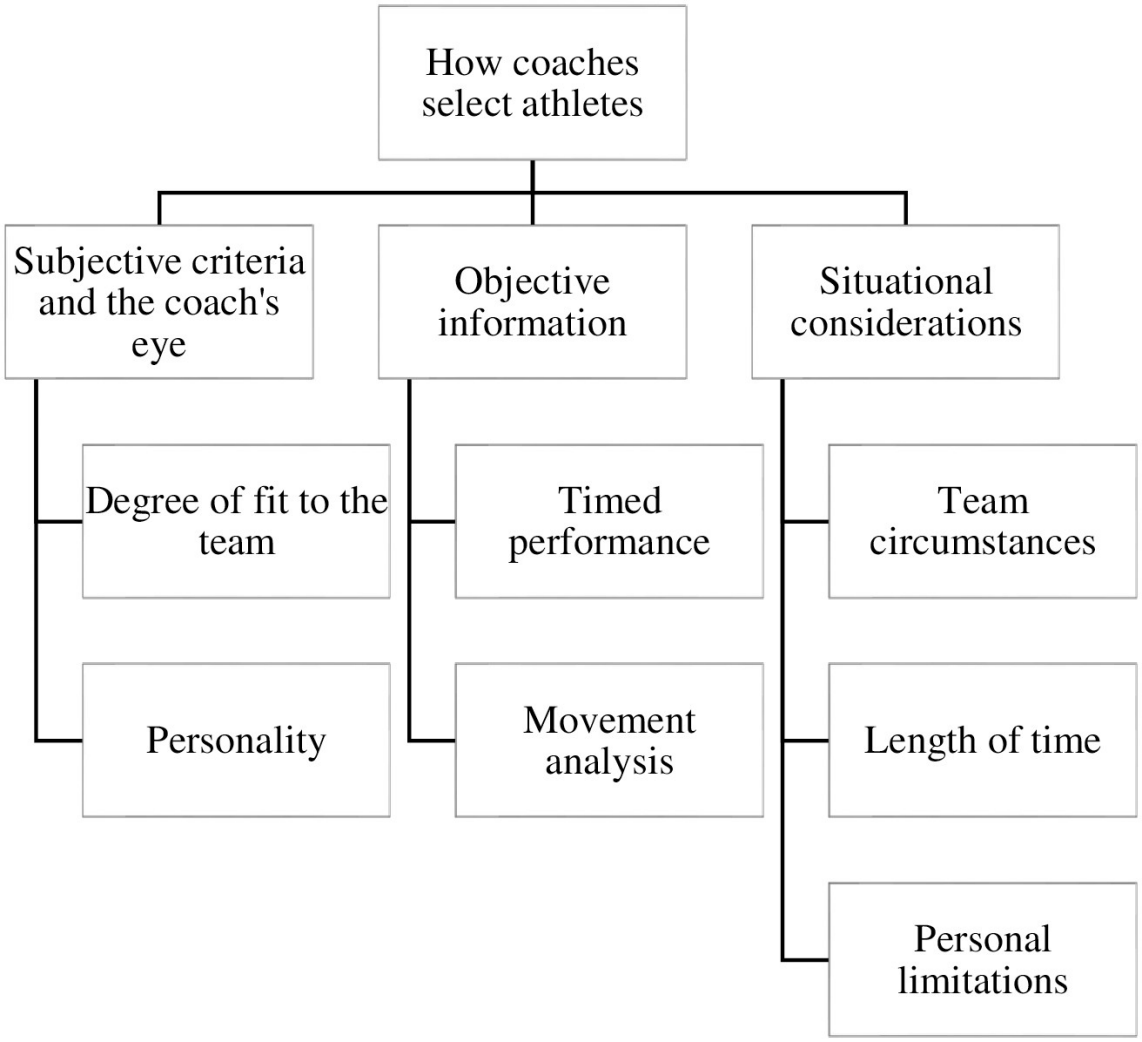
Organization of themes.

From this quote, it was clear his approach to selection was multifactorial as he listed a range of subjective and objective qualities, characteristics, traits, and abilities, demonstrating the scale and complexity of his identification and selection processes. The authors also recognized that upon sharing their lists of variables, certain quantities (e.g., number of years, metrics, times or values) were assigned to these characteristics, giving the idea that perhaps a mental model (i.e., an organizational structure of ideas and preferences) was being used in the decision-making process. Similar to Coach 1, other coaches offered a multi-step approach to selection. Upon further investigation and subsequent analysis, three commonly discussed themes were generated in relation to the information used for selection decisions including, a) subjective criteria and the coach’s eye, b) objective sources of information, and c) situational considerations. These three main themes are presented along with their associated sub-themes and supported by coach commentary below—for a visual depiction of the organization of themes and sub-themes, please see Fig 1.

### Subjective criteria and the coach’s eye

Our interview data indicated coaches recognize patterns using their ‘trained eye’ (known colloquially as the ‘coach’s eye’). Using the coach’s eye, it was interpreted they form judgements about an athlete’s past, present, and future ability. For example, Coach 10 expressed, “there’s some people that you just see them run for the first time and just the way they flow and their form you just look at them and you’re like, ‘oh my God’”. When asked to explain how the coach’s eye was developed/built/gained, Coach 1 explained it was “developed over years of experience of watching talent come through a pathway”, and Coach 4 expressed, “I think it’s just like anything if you do it a very, very, very long time, it just becomes more nuanced in your ability to ‘feel it’. So, I’ve just been through more, worked with more people, I think my read is better”.

Through follow-up questions and further probing, the interviews revealed two areas coaches primarily focus on when using their ‘eye’ including, a) the ‘fit’ of an athlete to the team, and b) an athlete’s personality, which will be explored as sub-themes below.

#### Degree of ‘fit’ to the team

Through discussions with coaches, one of the primary uses of their ‘eye’ was to observe whether an athlete is/was a good ‘fit’ to the team. For example, Coach 4 remarked, “I just tell people, you’re checking us out, but we’re checking you out, and the fit sometimes isn’t great”. This ‘fit’ was interpreted to be an important component in building a successful distance running team for this sample of coaches, which is perhaps surprising because of the perceived nature of the sport of distance running (i.e., as an ‘individual’ sport). However, based on the present discussions, the quality of the teams’ dynamics was mentioned frequently as an important consideration when making selections decisions. For instance, Coach 6, referenced his perception of another elite coach’s beliefs when considering team dynamics and selecting athletes for reasons of ‘fit’:

… she’s trying to figure out, alright this athlete’s great, but if this athlete’s going to be absolutely toxic to our culture, I’ll move on because … the culture of what we’re doing with the entire group for her is more important than like one superstar athlete that’s going to come in and upset the whole apple cart.

Coach 6’s comments draw attention to the importance of team culture and how a mismatch in ‘fit’ may disrupt the team’s dynamics. It was interpreted the coach (whom Coach 6 is referencing) prioritizes this ‘fit’ even over running performance, speaking to the weight this variable may have in the judgement and decision-making process.

To further unpack what constitutes a ‘fit’ to the team, Coach 8 explained, “…you can’t be a square peg and fit into a round hole. So for us, you need to fit into our culture”. Additionally, Coach 2 noted certain aspects of what the ‘fit’ entails when he shared, “…there was a, just wasn’t the right fit. The athlete wasn’t feeling confident in how her program was being implemented”. These examples provide support for the idea that the ‘fit’ is subjective in nature, meaning different things to different coaches. For example, it was interpreted that coaches 8 and 2 consider ‘fit’ to include the values, goals, preferences, and circumstances of the athlete, the coach and the team. Specifically, Coach 8 discussed a ‘fit’ with the alignment to the team’s culture, and for Coach 2, he drew attention to an athlete’s confidence in his/her/their program as being an important element of ‘fit’. It is possible there are some common traits between coaches in what characteristics make up ‘fit’, however the degree to which each characteristic is important may be unique to any particular coach and may also vary based on his/her/their circumstances at the time of the selection decision.

When asked how coaches examine this ‘fit’ during assessment opportunities, they revealed various strategies. In one example, Coach 6 described the close attention he paid to athlete interactions while away on a ‘team bonding’ excursion,

to me it’s just a series of conversations and it’s even sometimes … putting athletes in competitive situations as part of our recruiting visits that we used to do with our groups of athletes … we would use, on purpose, some kind of competitive situation that they didn’t even realize they were in, like we’d go bowling or we’d play pool … just watch them, see how competitive they are or aren’t.

Additionally, Coach 8 shared:

you sit and see in that group how are they fitting in to that group, are they in there, are they working together, are they doing stuff together, are they helping each other as they go through that process, and you figure it out and I think at that point, the kids need to figure out that “hey, I want to be part of this environment and the culture, or am I out?”

Based on this discussion, it was interpreted Coach 6 and 8 believed their ‘eye’ can ‘see’ elements of ‘fit’ such as social comfortability, leadership, competitiveness. Moreover, these comments shed light on the potential underlying personality traits coaches are looking for when observing an athlete’s ‘fit’, which will be discussed in the next sub-phase.

#### Personality

In addition to the coach’s eye being used to discern an athlete’s ‘fit’ to the team, some coaches in the present sample used their ‘eye’ to determine athletes’ personalities. For example, Coach 5 shared he was looking for the following characteristics: “be nice to people, not bullying anybody, and we really start looking at what type of character do they have? It’s more important at the beginning of picking them than it is how fast they run”. This quote, once again, sheds light on the weight that coaches may place on such ‘subjective’ variables, which are perhaps even more heavily weighted than the athlete’s speed.

This idea of personality being an important factor for athlete selection was evident in multiple other coach responses. For example, Coach 10 shared:

we’ve had athletes, who you know say “[Coach 10], I’m gonna go Uber to Walmart and get some ponchos for the girls” or something. You know?…That’s great. Or bringing snacks for everybody or, I mean, those are all little things, but you know, if there’s a bunch of things like that, that’s really helpful to the team dynamic right or you know, I don’t know the girl that braids all the other girls’ hair right or always brings the ribbons or you know, brings the face tattoos or whatever.

Similarly, Coach 3 discussed:

people who, without being asked, perform a leadership role within the group…They show up for non-sport related team events. So, they generally participate in the life of the program. Those could be tiebreakers as well because those people bring value to the program besides from their performances and over the longer term, they’ll actually help us be better.

It can be gleaned from Coach 3 and 5’s comments there are a range of indicators coaches look for to determine whether an athlete displays a desirable personality trait like kindness and initiative-taking. Quotes such as these may offer insight into not only the type of athlete the coach is looking to select, but also the type of culture and value system embedded in the coach’s program.

Another frequently mentioned personality trait coaches looked for was work ethic. For example, Coach 8 discussed how he observed an athlete’s work ethic, demonstrated in the following quote, “you may have kids that come in that may not display at lot of talent, but they have a good work ethic, and I think when you are in distance, hard work can go a long way”. Additionally, Coach 10, shared how he assessed work ethic: “by observing them in practices, how they do on their off days, like easy runs or how they work on the elliptical or whatever”, which shows the importance of demonstrating a strong work ethic beyond practice and competitive environments.

### Objective information

In addition to the subjective information gleaned through the coach’s eye, the use of objective information was found to be important information for coaches to use when selecting athletes. The objective information discussed by the coaches in the present sample was categorized in the following two sub-themes, a) timed performance, and b) fitness/movement testing.

#### Timed performance

Unsurprisingly, race time (speed) was mentioned as the main objective criteria used in athlete selection. Put succinctly by Coach 9, “the great thing about running is it’s relatively objective”. It was interpreted many of the coaches use time trials as a preliminary way to assess and remove athletes in the initial stages of selection. As noted in his example, Coach 3 explains, “so our first criteria would be head-to-head performances”, and similarly, Coach 8, noted, “….every athlete who’s on our roster, even if they’re returning, has to go through that time trial and that’s what we’ll use to select our roster”. The use of time trials and head-to-head racing highlights a particular advantage the sport of distance running has when it comes to ecological validity and representativeness of testing measures. Despite this advantage however, it could also provide a challenge to selection as athletes who are injured, sick, or who are having an ‘off day’ may be removed at early stages in the selection process. Coach 7 noted this and explained the way his program allows for ‘wiggle room’ during selection periods to help minimize the impact of this potential wrongful, early exclusion,

…the athletes have … to hit those standards on the team; however, we’re pretty flexible if they are within, you know, a certain percentage of the time and depending on how many people we are going to collect, we take everybody and then as I mentioned earlier, if you are a bit off the time, there’s an opportunity for you to make the team down the road.

A similar approach was mentioned by Coach 9 who explained, “I’ll base it off of what I see in practice over the week, that first week of classes, and I’ll temper it a little bit sometimes [when] I’ve got a really strong athlete coming in, but maybe the fitness, because of injury or illness, isn’t quite there”. Even at the highest levels of sport performance, it was interpreted coaches consider these variables when analyzing race times as noted by Coach 6:

…and Athletics Canada, this is all online, you can look on the selection criteria, the Canadian Athlete Performance Pathway or carding pathway for Athletics Canada called ‘CAAP’, and the majority of it is based on points that you get from your performances but there is a selection committee that comes together that looks at how you’re tracking, how you’re tracking compared to your team, whether you’ve had injury or illnesses, did you bring in reports, were you just pregnant?

As noted in this quote, there are particular organization-specific approaches that coaches consider, which ultimately may shape the way selections occur. This highlights an important area for future investigations, as the field could benefit from examining the policies and structures in place through various means (e.g., discourse analysis), to better understand the potential influence(s) on coaches’ decision-making practices.

#### Movement analysis

Another frequently mentioned source of information used for selection decisions was movement analysis. In this case, coaches and other support staff (like strength and conditioning coaches) judge, measure, and analyze athletes’ physical abilities using fitness testing and screening protocols as noted by Coach 2,

any athlete that comes into our environment has to go through their intake, and the intake looks like, you go down to S&C [Strength and Conditioning] and you go through a whole bunch of movements. It is through these movement screens that coaches are looking for indicators for running ability.

When asked to elaborate on how exercises and movements relate to running performance, Coach 2 explained:

it’s a bit of a screen, a preventative screen to see if there’s any issues we have to address before we can load them appropriately but it also gives us a lot of information as to sort of what we’re going to do in the weight room in order to get them [to] their best selves for whatever event group they’re in…They have a physio screen as well, so they go in a room with a one of the our physiotherapists and they go through a whole series of movements ranges and motion and stuff… The big thing we’re looking for is whether or not there’s symmetry, you know, if one side is bad. That’s what leads to injuries; we always want symmetry.

Similarly, Coach 7 shared, “if they [the athletes] show some agility, show some speed, show some strength, they show some flexibility, then those are things we’re going to be looking for as well”, further supporting the idea that physical (and perhaps more objective) indicators are considered when crafting judgements about an athlete’s present, and perhaps future, running ability. It can also be interpreted from these two quotes that movement screens can be a helpful tool to highlight an athlete’s risk of injury. Using Coach 2’s explanation, we can interpret symmetry and other physical indicators are important for considering an athlete’s likelihood to sustain an injury and, by extension, to try and determine what type of training would/should be provided for certain athletes in different circumstances.

### Situational considerations

In addition to the subjective and objective ways coaches obtain information, many coaches described internal (within-coach and within-team) and external (within program/sporting organization) considerations influencing their decision-making process for selection. These included a) team circumstances, b) length of time, and c) personal limitations, and are explored in depth below.

#### Team circumstances

It was evident coaches’ selection decisions considered the circumstances of the team in a number of different capacities. This gave the impression that coaches do not simply select the ‘best’ or most ‘talented’ performers. Rather, in the discussions with coaches, it was interpreted that many selections consider the range of ages and experiences (preferring a blend of senior and junior athletes), consider the balance of the number of graduating athletes with rookies (for coaches in a collegiate setting), and consider how a coach may be viewed and judged for the decisions he makes. For instance, Coach 1 noted, obviously, we’d look at our gaps too right? So, who’s on our team? Do we need to replace in year one, year two, year three of this athlete’s development and where do we think we can fit in? So, constantly looking at a little bit of a gap analysis to see where our athletes [are] now and where we need to backfill.

As described in his comment, Coach 1 considered the diversity of experience (measured by number of years in the program) within his team, which may speak to the specific role he has within the collegiate coaching environment. In particular, at the university-level, the number of athletes on a team stays fairly consistent from year to year, and thus, coaches try to fill positions with incoming athletes as other athletes leave the program for various reasons (graduation, eligibility requirements etc.).

In addition to making decisions regarding experience levels of athletes on the team, it was interpreted coaches consider how their decisions are viewed by others. Coach 10, for example, considered the type of message he was sending to the team by selecting certain players over others, as he explained,

… I hate the idea of someone being on the team for four years and then you know not letting them run their fifth year. I don’t think that’s really fair, … but of course if there’s a young athlete that’s coming in and has the ability to be much, much, much, better than where they are right now, you know, you want them to be part of your program …you’d also don’t want the young people to see oh, hey if you’re not, if you haven’t improved in year four and five, you get cut from the team either, right?

This contemplation speaks to the multiple social and political factors Coach 10 considered in his selection process. These considerations further highlight how complex and nuanced these selection decisions can be and draw attention to the potential risk involved with making such decisions (i.e., perception of social image, emotional cost etc.).

#### Length of time

In the present sample, coaches had varied responses to the length of time they were afforded to make selection decisions. For some coaches at the collegiate level, their windows for selection were described as being relatively short. This was noted by the coaches as a possible constraint due to the variability in how long it takes for an athlete to demonstrate his/her/their ‘potential’ in the sport of distance running. For example, when asked how long it takes Coach 4 to determine if an athlete has ‘what it takes’ to be a part of the team, he responded, “so the answer is somewhere between 15 seconds and five years”. Similarly, Coach 3 explained,

[Athlete A] is a good example, it’s where it took her … eighteen months before we really saw her potential. It was exciting to watch; she could do a lot, but you were never sure if it she was going to sustain it. But it became apparent after about a year to eighteen months that she was going to be the real deal.…Our sport’s famous for having people bloom late and do amazing things.

Coach 7 echoed these sentiments in his response: “it doesn’t happen very often, but I would say every other year I probably have one or two athletes I didn’t even know of, that showed up at our trials and made our team. So, [regarding] talent, sometimes you don’t even know about talent coming in”. Taken together, these commentaries help provide insight into some of the unique aspects of distance running. It also helps to highlight the high degree of variability and potential instability of an athlete’s performance, which draws attention again to the challenges faced by coaches (and other selectors) when forced to make selection decisions, especially early in an athlete’s life and within relatively short periods of time.

#### Personal limitations

It was surprising (yet humbling) to hear so many coaches recognized their own personal limitations, biases, and constraints when judging an athlete’s ability. For example, Coach 4 acknowledged his fallibilities and shared even after years of coaching—“I still haven’t figured it out!”. In another example, Coach 6 commented, “you’ve got that gut feel, sense check, and at times you are going to have some type 1 or type 2 errors, false positives and false negatives and you’re never going to get rid of that”. This specific reference to making errors (wrongfully selecting athletes and wrongfully rejecting athletes) along with Coach 4’s humility in not having mastered the selection process speak to an awareness of the ‘talent wastage’ in the sport of distance running. When asked why coaches believe this talent wastage in the form of selection errors continues to occur (in the form of a follow-up question), Coach 3 explained,

maybe there are factors beyond their [coaches] control involved like favouritism. You’re naturally going to get along better with some athletes on the team than others if you have something in common say for instance. I have club athletes who I have coached since grade eight or nine and I know them well, I know their parents in many cases and so if I were to select an athlete like that over someone else and didn’t have a clear criteria to justify that selection, it could look like I’m just playing favorites.

This quote offers a lot to unpack as it is rich in nuance, however, a key takeaway from this quote is the coach’s awareness of the potential influences of favouritism. It was interpreted Coach 3 tried to even mitigate the impact of this favouritism by creating and upholding a more objective selection criteria. An important area of future explorations will be to further explore the perceived influence of ‘favouritism’ and how it relates to final decisions being made for athlete selection.

## Discussion

The present research seeks to contribute to an underexplored area of study within sport sciences. Using a pragmatistic lens, the authors analyzed coaches’ perspectives of their experiences, processes, and procedures relating to athlete selection. Coaches discussed the various sources of information that ‘work’ and have ‘worked’ to craft judgments and make selection decisions. Through these discussions, a clear picture has been painted that coaches incorporated multiple strategies, practices, and approaches when assessing and selecting athletes to their teams. Our interpretations of the data support the idea that some coaches formed mental models of various preferences relating to the qualities, characteristics, skills, and abilities of athletes which are diverse and multidimensional in nature (i.e., utilize a combination of objective and subjective preferences). It was also interpreted coaches considered multiple environmental and situational conditions when using these mental models, indicating a relatively fluid, nuanced, and dynamic approach to athlete selection.

Perhaps the most surprising finding of the present study was the heavy reliance coaches placed on certain subjective preferences when selecting distance running athletes. While many coaches referenced their objective criteria (head-to-head racing (who beat whom), race performance (rankings), and time trials (speed)), nearly all discussed (and sometimes even prioritized) the ‘intangible’/subjective qualities for athlete selection. As evidenced by the various discussions surrounding an athlete’s ‘fit’ to the team and the alignment (with both the coach and the team more generally) of the athletes’ values, culture, personality, and beliefs, it indicates that coaches assign a significant weight to these ‘subjective’ aspects of athlete selection in their mental models. Specifically, coaches emphasized an athlete’s personality as an important feature to consider when making selections. That said, the responses offered such varied personality traits valued by the coaches, that a single, or even a list of, desirable trait(s) would be too complex to untangle from the rich discussions with this group of coaches. These findings are likely a result of the various environmental and societal factors at play (such as culture, gender identity, background, education, location etc.), as they are all likely to shape a coach’s preferences for something like personality traits. Conversely, it is possible the design of this study may be affected by the false belief that a coach has the ability to articulate something that may be perceived as ‘intuitive’. As Silver [61] notes, experts (in this case, coaches) often have a ‘feel’ (sometimes referred to as ‘gut feeling’ or intuition; for an example see Roberts et al., [62]) for what they want to ‘see’ when it is easily observable. When these traits/characteristics/skills are more difficult to judge, however, (like an athlete’s ‘potential’), it can be easily overlooked, misjudged, and/or miscommunicated. Moreover, it should be noted, this heavy reliance of subjective values may be a unique finding of the present sample of coaches and their environments (collegiate and national/international level competition), or it may speak to some of the unique elements of the sub-culture of distance running (i.e., dedication to training, tolerance for pain, ability to endure repetitive training) as noted in the work by Allen Collinson and Hockey [32]. Either way, future investigative work extending beyond ‘surface-level’ interpretations (e.g., deeper approaches than QD), would be beneficial to gain a more robust understanding of selection practices.

Interestingly, this focus on subjective skills/traits/characteristics does not necessarily align with the majority of research in TID in sport [3,7]. Rather, most of the research conducted to date has focused on the physical and physiological profiles of athletes. For instance, research conducted on elite endurance athletes (including distance runners) has highlighted physiological [63,64], biomechanical [65,66], and genetic factors [67–69], but relatively little is known about the various psychological traits (for exceptions see [70–72]). This could be due to a multitude of reasons, namely the potential difficulty in determining which ‘softer’ skills to investigate, and which of the various methods (and combination of methods) could and should be used to explore such skills (e.g., questionnaires, interviews, tests, etc.). It is also possible coaches already have measures in place to assess the preferred traits and characteristics (which may be ‘seen’ using the coach’s eye), but current assessments and measures have yet to capture this nuanced and complex tacit knowledge. Future work could seek to explore this tacit knowledge in rigorous and ecologically-valid ways which could further enhance coach education strategies and help highlight the potential strengths and weaknesses of such approaches.

Perhaps a less surprising finding was the frequent mention of injury by the coaches. Specifically, coaches expressed their awareness of potential indicators for injury, recovery strategies for overcoming injury, and ability to tolerate injury, indicating a particular value placed on this specific component in their selection criteria. This is in strong alignment with the research surrounding the sub-culture of distance running that embraces pain and discomfort [32,73]. Not only does this finding shed light on the coaches’ selection preferences, it also sheds light on the coaches’ subjective beliefs of running talent, which may in turn, cyclically inform their selection decisions. In work by Allen Collinson and Hockey [32], the authors used a symbolic interactionist lens to analyze the impact of long-term injury on the identities of two middle- and long-distance runners. The authors note (along with others like Pike and Maguire [74]) that ‘distance running incorporates pain and injury as routine and normalized features’ (p. 388). These injuries, however normalized, present a serious risk towards training, performance, well-being and athlete identity [32,34,75,76]. It is likely a reason why coaches consider injury, potential to injure, and the ability to recover from injury, as important components in their selection considerations.

Our findings presented mixed results on whether judgements around injury and potential for injury were indicators selected for, or against. In other words, this is not necessarily to say having (or previously having) an injury is a reason for de-selection. Some researchers have explored whether injury was potentially beneficial in some ways, as there may be developmental benefits from both a physiological and mental perspective [73,77–79]. Specifically, their work Bluhm and Ravn [73] examined how an understanding of running-related pain and injury may contribute positively and in a meaningful way to the psychology of competitive and serious distance runners. As mentioned, however, the findings from the present investigation were unclear whether these ‘features’ are routinely selected for or against, just rather were found to be considerations in the selection process.

What was particularly interesting in the current investigation was coaches’ awareness of their own limitations and potential errors in selection processes. The coaches in this study recognized they were susceptible to such errors and referenced the strategies they adopted to try and mitigate the effect of their biases and mental short cuts. This mindset and ability to question beliefs and challenge practices is a quality often regarded as a superior strategy to enhance decision-making accuracy [80–82]. A strategy that deserves further investigations, however, is the reliance/use of the coach’s eye. To date, the specific elements of what the coach’s eye is ‘seeing’, along with its value in decision-making accuracy remains relatively unknown. It is often believed the coach’s eye *combined* with objective testing data can help increase the accuracy in selection decisions [83–85]; however, more work is needed to confirm this across sports and competitive levels. On the one hand, this subjectivity in the coach’s eye can be useful in athlete identification as coaches often have many years of experience, multiple levels of standardized training, and often a background in competitive sport participation. On the other hand, evidence from sport science and other disciplines (such as economics and medicine) suggests subjective approaches to selection can be biased, error-filled, and costly [61,86,87]. A blended approach of integrating both subjective (e.g., coach’s eye) and objective (e.g., metrics) likely holds the greatest value to a coach when making decisions about an athlete’s likelihood of future success. The relative contribution of each, however, is something still to be discovered.

## Limitations

Despite the potential advancements this study makes for understanding distance running coaches’ sources of information for selection decisions, there are a number of limitations to consider. One of the most notable lies in the way the data were collected. It is possible more representative information could have been gained through different data collection strategies. It has been recognized investigations into coach decision-making (including the present study) are divorced from real-world situations and it can be argued this approach changes the representativeness, relevance, and implications of the findings [88–90]. Specifically, the present study investigated what coaches believe their practices are, where a separate study could be conducted on what coaches do in real-time through various research designs. As noted by Roberts [18], the reflective nature of interviews may influence a coach’s perception of his/her/their processes, including his/her/their justification for previous selection decisions, presenting a notable limitation. Therefore, information gleaned from the present study should be interpreted and considered only within the context in which the information was gathered, analyzed, and presented. Specifically, the current sample is only reflective of a small sub-sample of the coaching population (at one point in time, from all white, male coaches, and from Canada).

Ideally, future work could shift from a reliance on recall-based strategies and towards capturing real-time decision-making where possible. One alternative or complementary approach for researchers to consider is the ‘think aloud method’ where participants provide verbal reports of their thoughts allowing for a ‘live’ look at the mental processes of decision makers (for examples of this method in research, see Eccles et al., [91] and Whitehead et al., [92], and specifically in distance running, see Samson et al., [72]). For exploring similar research questions to those examined in the present study, think aloud protocols may help unpack what the coach’s eye is seeing in live time and would offer richer insights into coaches’ mental modelling and selection considerations, thus increasing the validity and representativeness of the data. The approach itself, however, presents some notable administrative constraints such as time (selections can happen over days, weeks, or months), resources (many coaches may be involved meaning many hours of transcripts to analyze), proprietary information (sensitive information or coaches protection over their selection practices), and logistics (multiple locations and days).

Another limitation lies within the nature of interviewing itself, as multiple factors may affect the quality and quantity of the data. Some of the more notable and common biases include the hindsight bias, (also known as the narrative fallacy), recency bias/recollection bias [93], and social desirability bias [94]. In the case of hindsight bias, respondents may recall certain situations and occurrences from their past and use them to create a more congruent story/narrative of facts that may not have been connected. For example, if a coach remembers a certain athlete who did well, he/she/they may incorrectly remember qualities that fit a certain profile or stereotype, or that may inflate certain attributes that may not have been connected. This may lead an interviewee to misremember or inaccurately report his/her/their experiences, may embellish stories, and may miss critical pieces of his/her/their narrative. Similarly, the recency bias/recollection bias may lead an interviewee to only draw from the most recent memories whereby stories shared may be only representative of more current states, rather than a wholistic perspective over time. For example, if a coach has recently completed a selection process, he may rely on that singular experience to reflect upon as it is more easily retrievable in his/her/their memory [93]. To help mitigate the effect of the recollection bias, a probing question “how do you think your current approach to talent selection differs from the way you selected players in previous years?” was added to the interview guide.

Finally, some coaches may have withheld information that could have been understood to be proprietary in nature. Only one coach noted this outright and explained that he could not provide all the details on his selection criteria in an effort to maintain his teams’ competitive edge. This could mean the data presented here are incomplete, generalized, or superficial compared to what is experienced in reality. While this mindset and approach to information protection is commonly practiced (and understandably so), it may continue to act as a barrier to understanding elite coaches’ tacit knowledge, and subsequently, improvements in athlete selection.

## Conclusion

The present study highlights the multidimensional approaches used by the present sample of coaches to gather information, form judgements, and make selection decisions. The coaches in the present sample were believed to rely on their coach’s eye to gain information on subjective characteristics such as an athlete’s ‘fit’ to the team and an athlete’s personality. Coaches also utilize objective information gained from head-to-head performances and other race times along with movement analyses. When making decisions regarding athlete selection, coaches also consider the circumstances for the team, including the range of years of experience, number of years left for eligibility reasons and the ‘gaps’ of the team. Last, coaches have an awareness of their cognitive and systemic constraints such as the length of time needed to make a decision, personal biases, and errors in judgement.

This research has implications for coaches, sport organizations and talent identification and selection programs alike. Findings of this investigation may act as a launch pad for future qualitative and quantitative research on the different sources of information utilized by coaches (e.g., personality traits). The knowledge gained from this research may also be used in coach education and development, highlighting the ways elite coaches assess, evaluate, and form judgements about athletes.
